# Imaging PARP Upregulation with [^123^I]I-PARPi SPECT/CT in Small Cell Neuroendocrine Carcinoma

**DOI:** 10.2967/jnumed.123.266348

**Published:** 2024-04

**Authors:** Honest Ndlovu, Ismaheel Lawal, Kgomotso Mokoala, Dineo Disenyane, Nonhlahla Nkambule, Sheynaz Bassa, Yonwaba Mzizi, Meshack Bida, Mike Sathekge

**Affiliations:** 1 Nuclear Medicine Research Infrastructure; Department of Nuclear Medicine, Steve-Biko Academic Hospital, University of Pretoria, Pretoria, South Africa;; 2 Department of Radiology and Imaging Sciences, Emory University, Atlanta, Georgia;; 3 National Health Laboratory Services and University of Pretoria, Pretoria, South Africa; and; 4 Department of Radiation Oncology, University of Pretoria and Steve Biko Academic Hospital, Pretoria, South Africa

A 61-y-old man underwent [^18^F]FDG PET/CT for staging of right-lung small cell neuroendocrine carcinoma. The images (Fig. 1A) showed a tracer-avid right-lung mass. As part of a feasibility study approved by the University of Pretoria Ethics Committee, the subject gave written informed consent to additionally undergo [^123^I]I-poly(adenosine diphosphate ribosyl)-polymerase (PARP) inhibitor (PARPi) SPECT/CT. To the best of our knowledge, this study is the first in humans to use [^123^I]I-PARPi to image PARP upregulation in solid tumors. Images acquired at 4 h after tracer administration demonstrated [^123^I]I-PARPi avidity in the right-lung mass. Subsequent [^18^F]FDG PET/CT imaging obtained for response assessment after 2 cycles (Fig. 1B) and 6 cycles (Fig. 1C) of chemotherapy with carboplatin and etoposide continued to show the lung mass, along with new metastases in the mediastinal nodes, right adrenal gland, liver, and bone, consistent with disease progression. Immunohistochemistry was done using rabbit antibody against PARP, with splenic sinusoids and tonsillar tissue as negative and positive controls, respectively. On the baseline right-lung biopsy sample, immunohistochemistry was strongly positive for PARP upregulation.

**FIGURE 1.**
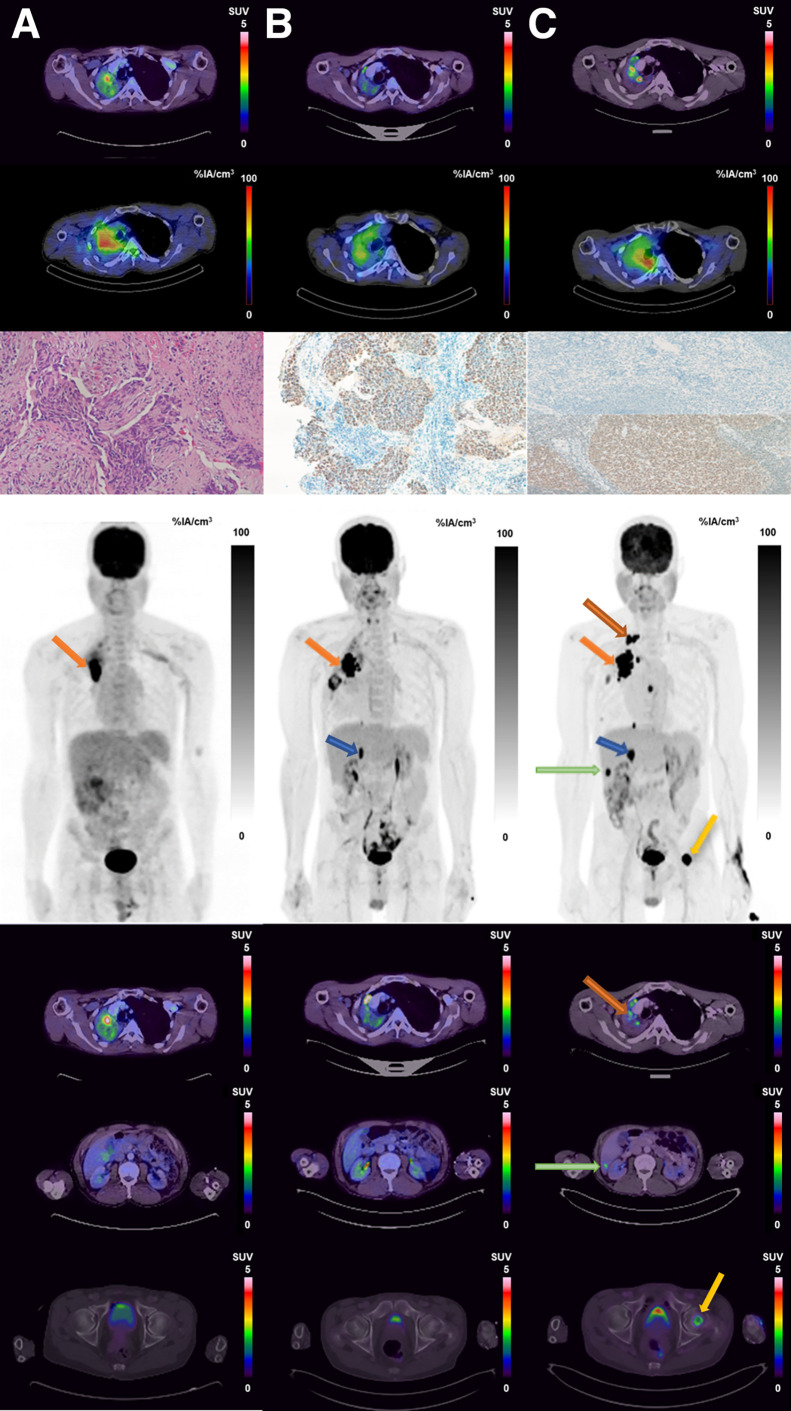
Imaging at baseline (A), early response assessment (B), and end of therapy (C). Shown from top to bottom are transaxial PET/CT, transaxial 4-h SPECT/CT, immunohistochemistry (×20 hematoxylin and eosin staining at left, ×10 PARP staining at middle and right), maximum-intensity-projection PET, and PET/CT. Hypermetabolic lung mass is seen on PET/CT (SUV_max_, 3.8) and SPECT/CT at baseline. Uptake on both modalities persists at early response assessment and at end of therapy (SUV_max_, 4.5 and 4.9, respectively). Immunohistochemistry was strongly positive for PARP upregulation. Disease progression was noted on maximum-intensity projections and on PET/CT, with new lymph node metastases indicated by amber arrow above orange arrow seen on both maximum-intensity projection and PET/CT imaging (SUV_max_, 4.5), adrenal gland metastases indicated by blue arrow on maximum-intensity projection images, liver metastases indicated by green arrow on both maximum-intensity projection and PET/CT images, bone metastases indicated by yellow arrow in the maximum-intensity projection images and PET/CT images (last row, third column). %IA = percentage injected activity.

PARP enzymes are upregulated after single-strand DNA breaks as part of the DNA damage response. Their upregulation confers resistance to conventional therapies but makes treatment with PARPi feasible. PARPi therapy is a novel option with survival benefits in tumors exhibiting homologous recombination DNA repair deficiencies (1). Without appropriate patient selection, the benefits of PARPi therapy are limited. These can be improved by selecting patients through use of immunohistochemistry and whole-body imaging for PARP upregulation (2,3). The synthesis and preclinical characterization of [^123^I]I-PARPi have been previously reported (4). Here, we report the feasibility of [^123^I]I-PARPi SPECT/CT as a noninvasive tool for whole-body assessment of PARP upregulation in lung small cell neuroendocrine carcinoma. A potential application is patient selection and response prediction for PARPi therapy. [^123^I]I-PARPi may have therapeutic use by harnessing the cytotoxic effect of the emitted Auger electron.

## DISCLOSURE

Theragnostic Inc. supplied the PARPi cold kit, iThemba Labs supplied [^123^I]iodine, and AXIM Nuclear & Oncology perfomed the radiolabeling. No other potential conflict of interest relevant to this article was reported.
